# Lower Body Weight in Rats Under Hypobaric Hypoxia Exposure Would Lead to Reduced Right Ventricular Hypertrophy and Increased AMPK Activation

**DOI:** 10.3389/fphys.2020.00342

**Published:** 2020-04-20

**Authors:** Karen Flores, Patricia Siques, Julio Brito, Stefany Ordenes, Karem Arriaza, E. Pena, Fabiola León-Velarde, Rosario López, Ángel L. López de Pablo, Silvia Arribas

**Affiliations:** ^1^Institute of Health Studies, University Arturo Prat, Iquique, Chile; ^2^Institute DECIPHER, German-Chilean Institute for Research on Pulmonary Hypoxia and Its Health Sequelae, Iquique, Chile; ^3^Department of Biological and Physiological Sciences, Facultad de Ciencias y Filosofía/IIA, Cayetano Heredia University, Lima, Peru; ^4^Department of Preventive Medicine and Public Health, University Autónoma of Madrid, Madrid, Spain; ^5^Department of Physiology, Universidad Autónoma de Madrid, Madrid, Spain

**Keywords:** right ventricle hypertrophy, hypobaric hypoxia, AMPK, body weight, high altitude

## Abstract

**Background:**

Both chronic hypoxia (CH) and long-term chronic intermittent hypoxia (CIH) exposure lead to right ventricular hypertrophy (RVH). Weight loss is an effective intervention to improve cardiac function and energy metabolism in cardiac hypertrophy. Likewise, caloric restriction (CR) also plays an important role in this cardioprotection through AMPK activation. We aimed to determine the influence of body weight (BW) on RVH, AMPK and related variables by comparing rats exposed to both hypoxic conditions.

**Methods:**

Sixty male adult rats were separated into two groups (*n* = 30 per group) according to their previous diet: a caloric restriction (CR) group and an ad libitum (AL) group. Rats in both groups were randomly assigned to 3 groups: a normoxic group (NX, *n* = 10), a CIH group (2 days hypoxia/2 days normoxia; *n* = 10) and a CH group (*n* = 10). The CR group was previously fed 10 g daily, and the other was fed ad libitum. Rats were exposed to simulated hypobaric hypoxia in a hypobaric chamber set to 428 Torr (the equivalent pressure to that at an altitude of 4,600 m above sea level) for 30 days. Measurements included body weight; hematocrit; serum insulin; glycemia; the degree of RVH (Fulton’s index and histology); and AMPK, mTOR, and PP2C expression levels in the right ventricle determined by western blotting.

**Results:**

A lower degree of RVH, higher AMPK activation, and no activation of mTOR were found in the CR groups exposed to hypobaric hypoxia compared to the AL groups (*p* < 0.05). Additionally, decreased glycemia and serum insulin levels were observed. Interestingly, PP2C expression showed an increase in the AL groups but not in the CR groups (*p* < 0.05).

**Conclusion:**

Maintaining a low weight before and during exposure to high-altitude hypoxia, during either CH or CIH, could prevent a major degree of RVH. This cardioprotection would likely be due to the activation of AMPK. Thus, body weight is a factor that might contribute to RVH at high altitudes.

## Introduction

Exposure to hypoxic environments under both chronic hypoxia and long-term chronic intermittent hypoxia induces cardiopulmonary changes that allow the maintenance of the circulatory demands and the homeostasis of tissues under conditions of limited oxygen availability (Ostadal et al., 1998). Under Long-term chronic intermittent hypoxia condition individuals work at a high altitude for days and rest at sea level for the same period (Richalet et al., 2002). Hypoxia exposure elevates pulmonary artery pressure by vasoconstriction (Von Euler and Liljestrand, 1946; Moudgil et al., 2005), and after a long period of exposure there is a remodeling of pulmonary vasculature leading to right ventricular hypertrophy (RVH) (Brito et al., 2007, 2018; Penaloza and Arias-Stella, 2007).

Cardiac hypertrophy is associated with increased cardiomyocyte cell volume, enhanced protein synthesis, and changes in gene transcription and translation (Glennon et al., 1995). Additionally, human studies and animal experimental models have identified that right ventricular dysfunction due to pressure overload is associated with metabolic derangements (Ryan and Archer, 2014). Because of its particular role, the heart requires high metabolic activity due to constant overloading (Noppe et al., 2014).

AMP-activated protein kinase (AMPK) is a heterotrimeric protein kinase composed of a catalytic α subunit and two regulatory subunits (β & ɤ). As an intracellular energy sensor, in the heart, AMPK is activated in response to an increase in the AMP/ATP ratio under stress conditions such as hypoxia, hypertrophy and hypoglycemia (Arad et al., 2007; Viollet et al., 2009). Studies in mice revealed that AMPK activation attenuates the development of cardiac hypertrophy by inhibiting protein synthesis and activating autophagy (Chan et al., 2004, 2008; Kang et al., 2011; Li et al., 2014). AMPK activation can also increase the uptake of glucose, enhance fatty acid oxidation (Dyck and Lopaschuk, 2006; Nagendran et al., 2013) and inhibit protein synthesis to reserve energy stores (Liu et al., 2006). Therefore, AMPK would have a protective role by restoring the energy balance and a key role against cardiovascular diseases and cellular stress (Dolinsky and Dyck, 2006). This role has been seen in humans under chronic hypoxia (CH) (Zhang et al., 2018) but has rarely been studied in hypobaric chronic intermittent hypoxia (CIH). The cardioprotective role of the AMPK pathway against cardiac hypertrophy involves mammalian target of rapamycin (mTOR), which is a major regulator of myocardial protein synthesis and a major driver of cardiac hypertrophy (Proud, 2004) whose activation regulates cell proliferation, apoptosis, cell migration and metabolism (Li et al., 2014). However, the inactivation of AMPK by dephosphorylation has been described to be attributed to PP2C (Davies et al., 1995; Marley et al., 1996; Steinberg, 2007). PP2C is a protein serine/threonine phosphatase that controls the specific dephosphorylation of thousands of phosphoprotein substrates (Shi, 2009). The primary function of PP2C appears to be the regulation of stress signaling, although it also plays a role in cell differentiation, growth, survival, apoptosis, and metabolism (Lu and Wang, 2008).

The impact of caloric restriction (CR) on inducing weight loss in cardiac hypertrophy has substantial clinical importance (Karwi et al., 2019). Recently, it has been reported in both humans and animal models that overweight and obesity influence high-altitude illness outcomes such as pulmonary hypertension (San Martin et al., 2017; Brito et al., 2018). Studies in mice show that CR is a potent dietary intervention to produce beneficial cardiac effects (Kobara et al., 2015; Melo et al., 2016) through AMPK activation, which plays an important role in cardioprotection (Shinmura et al., 2005, 2008; Chen et al., 2013).

The impact of a change in body weight before and during exposure to hypoxia at a high altitude, as well as the activation of AMPK in RVH, is not well known. Therefore, it is hypothesized that during high-altitude hypobaric hypoxia, a lower body weight would promote a reduced RVH through the activation of AMPK. Thus, the aim of this study was to determine the influence of body weight (BW) on RVH, through caloric restriction compared to an ad libitum food intake regimen, as well as its association with AMPK and related variables, by comparing rats exposed to both hypoxic conditions (CIH and CH).

## Materials and Methods

### Animal Model

The model used for this experiment was largely described and validated previously in several studies (Siques et al., 2006). The study was performed on sixty male Wistar rats (12 weeks of age) obtained from the animal facility of the Institute of Health Studies of Arturo Prat University, Iquique, Chile. The rats were assigned to two groups according to the amount of food provided during the previous month of exposure (CR 10 g and AL daily). Then, it was obtained a CR group (body weight 251.6 ± 1.9 g; *n* = 30), which received 10 g/day of food (Corresponding to caloric restriction 70%), and an ad libitum (AL) group (body weight 434.6 ± 5.9 g; *n* = 30). This model of caloric restriction is based on the works in rats of Kobara et al. (2015) and Melo et al. (2016). Then, both groups were randomly divided into three groups: (1) a normobaric normoxia (NX) group (*n* = 10), which served as a sea-level control; (2) a chronic intermittent hypobaric hypoxia (CIH) group (*n* = 10), which underwent 2 days of exposure to hypobaric hypoxia alternating with 2 days of exposure to normobaric normoxia; and (3) a chronic hypobaric hypoxia (CH) group (*n* = 10), which underwent permanent exposure to hypobaric hypoxia. All groups received water ad libitum and a standard balanced diet for laboratory rats (22.0% crude protein, 5.0% crude fat, 5.0% crude fiber, 9.0% ash and 12% moisture (5POO^®^, LabDiet^®^, Prolab RMH3000). Food intake was measured through the determination of the amount of residual food, and fasting times were accurately controlled. The exposure time of each group was 30 days, and hypobaric hypoxia was simulated in a chamber at 428 Torr (equivalent to an altitude of 4,600 m above sea level). The time of ascension from sea level to 4,600 m above sea level was 60 min.

The chamber conditions were as follows: internal flow of 3.14 L/min of air and humidity between 21 and 30%. NX groups were located in the same room at sea level (760 Torr) and housed under the same chamber conditions as the groups exposed to hypoxia. The rats were placed in individual cages at a temperature of 22 ± 2°C and a circadian rhythm of 12 h of light and 12 h of dark. Movement inside the cage was not restricted, but no exercise was performed. At the end of the exposure period, the rats were euthanized with an overdose of ketamine (0.9 mg/kg of weight), organs were collected and stored at −80°C, and specific variables were measured.

The animal protocol and experimental model were in accordance with Chilean Law No. 20380 regarding animal experimentation and were approved by the Research Ethics Committee of Arturo Prat University, Iquique, Chile.

### Body Weight, Hematocrit, Blood Glucose, and Serum Insulin

Both biochemical and physiological parameters in all study groups were measured at day 0 under basal normoxic conditions and after 30 days immediately after removal from the chamber. The body weight and residual food were measured using an electronic scale (Acculab V-1200^®^, Chicago, IL, United States).

Blood extraction (1 mL) for biochemical measurements was performed via cardiac puncture under anesthesia (0.3 mg/kg body weight) after 10 h of fasting. The hematocrit (Hct) values, calculated as percentages, were measured using capillaries, which were centrifuged (5804 R Eppendorf AG^®^, Hamburg, Germany) at 5,000 rpm for 5 min. Glucose in blood was measured using a glucometer (CarenSensN^®^), and serum insulin was measured using a commercial kit (Reta Insulin ELISA Kit^®^, ALPCO, Salem, VT, United States).

### Western Blot Analysis

For protein analysis, 50 mg of right ventricular cardiac muscle was obtained from each rat. Protein extraction was started by tissue homogenization (Stir-Pak^®^, Barrington, IL, United States) with 500 μL RIPA lysis buffer, which contains a mixture of phosphatase and protease inhibitors (4 mM PMSF, 10 μM leupeptin, 1 mM EDTA, 1 mM EGTA, 20 mM NaF, 20 mM HEPES and 1 mM DTT). Then, the homogenates were centrifuged (5804 R Eppendorf AG^®^, Hamburg, Germany) at 12,000 rpm for 20 min at 4°C, and the supernatant was extracted. For the quantification of the total protein extracted, the Bradford reaction was used (Bradford, 1976) with a BioPhotometer (Eppendorf AG^®^, Hamburg, Germany) at 590 nm, and samples were then stored at −80°C. For western blotting, the samples were previously diluted with 2X Laemmli buffer [0.125 M Tris-HCl, 4% SDS (p/v), 20% glycerol (v/v), 0.004% bromophenol blue, 10% β-mercaptoethanol (pH 6.8)]. Then, 50 μg of the protein were separated according to their molecular weight (MW) under an electric field via sodium dodecyl sulfate-polyacrylamide gel electrophoresis (SDS-PAGE) (30% bis-acrylamide (v/v), 150 mM Tris (pH 6.8 and 8.8), 1.0% TEMED (w/v), H_2_O) at 6 and 12%. Electrophoretic separation was initiated with the application of direct current to 150 V over 80 min with a power supply (PolyScience^®^, EPS-300, Taipei, Taiwan, China), and the proteins were then transferred from the polyacrylamide gel to a polyvinylidene difluoride (PVDF) membrane at 180 mA for 90 min with a semidry electroblotting system (Owl^TM^ HEP systems, Thermo Fisher Scientific^®^, United States).

To avoid nonspecific antibody binding, the PVDF membrane was blocked with bovine serum albumin (BSA) at a concentration range of 3–5% in TBS-T solution containing 10 mM HCl, 150 mM NaCl, and 0.05% Tween-20 at pH 7.4. The blocking time was 1 h at room temperature. Then, the membrane was incubated with the corresponding primary antibodies [AMPKα1/2 (sc-25792), p-AMPKα1/2 (sc-101630), mTOR (sc-517464), p-mTORC1 (sc-293133), PP2Cα (sc-517264), and β-actin (sc-130657)] at a dilution of 1:500 (Santa Cruz Biotechnology^®^, Santa Cruz, CA, United States) and incubated overnight at 4°C. Finally, the membrane was incubated with secondary antibodies (anti-goat, anti-rabbit and anti-mouse antibodies, Santa Cruz Biotechnology^®^, Santa Cruz, CA, United States) at a dilution of 1:2,000 in 3% BSA for 1 h at room temperature, washed with TBS-T and then imaged in a dark room with a chemiluminescence kit (Chemiluminescence West Pico^®^, Super Signal Substrate, Thermo Fisher Scientific^®^, Rockford, IL, United States). The density of the bands was measured with ImageJ software and normalized according to β-actin expression and Ponceau staining. The activity levels of AMPK and mTOR were determined by the ratio of phosphorylated protein to total protein.

### Right Ventricular Hypertrophy and Histology

RVH was evaluated at the end of the exposure period (day 30) using Fulton’s index [RV/LV+Septum (g/g)], as described previously (Kay, 1980). Also, total ventricles weight vs. body weight ratio was obtained (g/g). For morphological assessment under light microscopy, the ventricular tissue was cut transversally and fixed in 4% paraformaldehyde at room temperature overnight and then dehydrated and embedded in paraffin. Paraffin-embedded tissue slices (5 μm thick) were routinely stained with hematoxylin and eosin (H&E) and finally, the area was measured through ImageJ software.

### Data Analysis

All data recorded were included in a database and analyzed using the SPSS program (IBM SPSS^®^ V.21.0^®^, Armonk, NY, United States). The normality of the variables was established by the Kolmogorov-Smirnov test, and all variables had a normal distribution. The means and standard errors (SEs) were calculated for all variables. To determine differences in the measured variables over time, a paired-sample Student’s t test was performed between the two groups. To establish the intergroup differences, repeated-measures analysis of variance (ANOVA) was used. For variables measured once, an independent Student’s t test and one-way ANOVA followed by the least significant difference (LSD) *post hoc* test were performed. The level of significance was established at the 95% confidence level, with *p* < 0.05 considered indicative of significance.

## Results

### Body Weight, Hematocrit, Blood Glucose, and Serum Insulin

**FIGURE 1:**
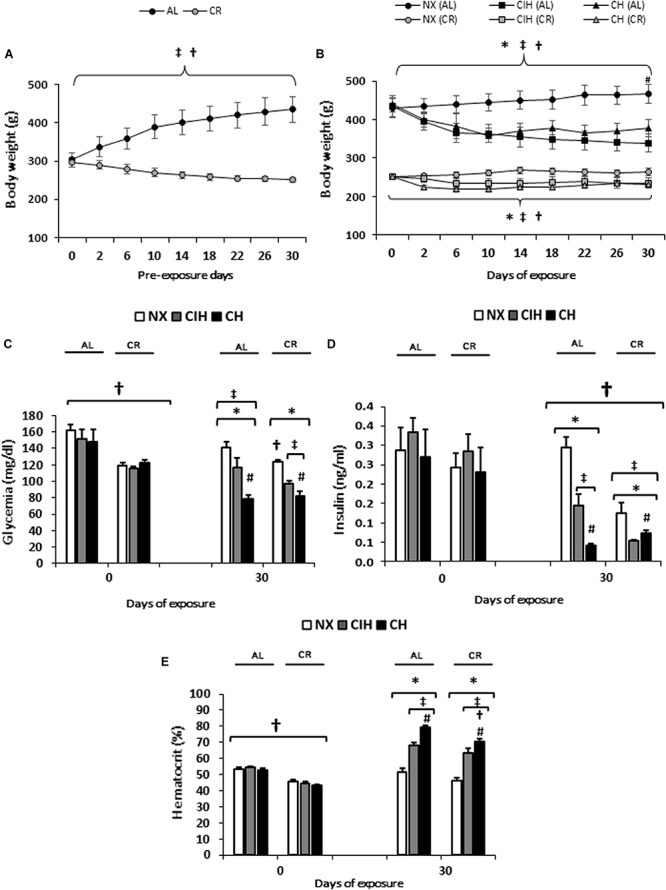
Physiological and biochemical parameters: Ad libitum (AL) and caloric restriction (CR) groups on days 0 and 30: normoxic group (NX), chronic intermittent hypoxia group (CIH), and chronic hypoxia group (CH). **(A)** Body weight (g) previous to exposure, **(B)** Body weight (g) during exposure, **(C)** Blood glucose (mg/dl) and **(D)** Serum insulin (ng/ml) **(E)** Hematocrit (%). The values are the mean ($\bar{\text{x}}$) ± standard error (SE). ^∗^*p* < 0.05: hypoxia-exposed vs. NX; ^#^*p* < 0.05: CIH vs. CH. ^†^*p* < 0.05: AL vs. CR; ^‡^Day 30 vs. Day 0 for each group NX, CIH and CH of AL and CR groups.

Previous exposure body weight was decreased in CR compared to AL group (*p* < 0.001) (Figure 1A). The body weight was lower in both the AL and the CR hypoxia-exposed groups (CIH and CH) than in the NX group (*p* < 0.001) at the end of exposure day 30 (Figure 1B). Remarkably, the AL group lowered their weight by a greater proportion than the CR group (*p* < 0.001). Importantly, food intake under CH in both the AL and CR groups was similar (10 g), whereas under CIH, both groups showed reduced intake, with less intake in the AL group than in the CR group while they were in the chamber. Interestingly, CR group under chronic hypoxia did not further reduce the food intake. Despite this observation, the AL group showed a higher weight than the CR group at the end of the exposure.

Both the AL- and CR-exposed groups exhibited a decrease in blood glucose levels and serum insulin compared to the NX groups and basal levels (*p* < 0.01). Interestingly, the CR group exposed to CIH showed lower insulin levels than the AL group exposed to CIH (*p* < 0.01) (Figures 1C,D).

Under hypobaric hypoxic conditions (CIH and CH), both the AL and CR groups showed an increase in Hct compared to the NX groups and basal levels (*p* < 0.001), with values being higher in the CH groups (*p <* 0.01). Notably, this increase was less in the CR groups than in the AL groups exposed to CH (*p* < 0.01) (Figure 1E).

### Right Ventricular Hypertrophy

**FIGURE 2:**
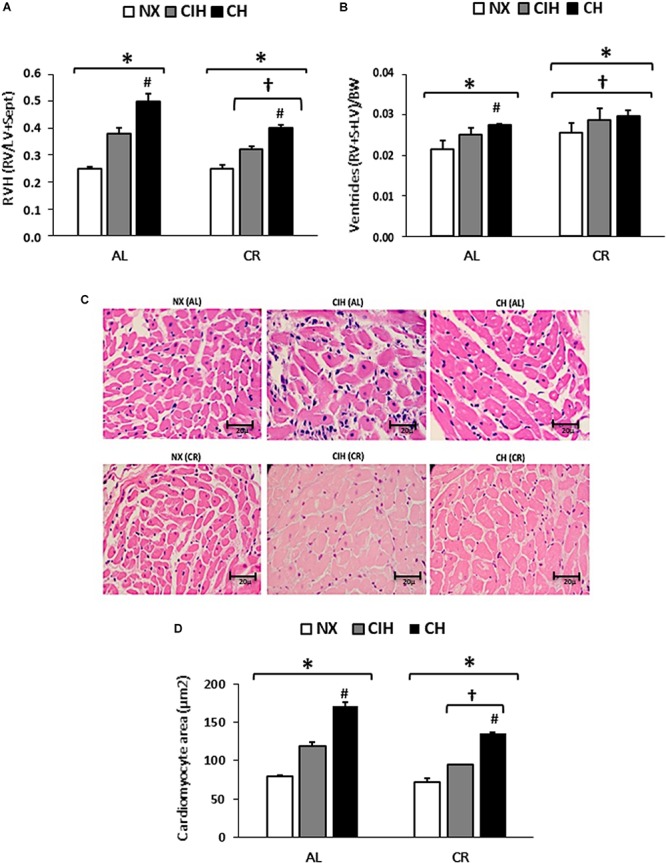
Grade of right ventricular hypertrophy (RVH): Ad libitum (AL) and caloric restriction (CR) groups on day 30: normoxic group (NX), chronic intermittent hypoxia group (CIH), and chronic hypoxia group (CH). **(A)** Expressed as Fulton’s index [right ventricle (RV) weight/(left ventricle (LV) weight + septum weight)]. **(B)** Ventricles (RV+S+LV)/BW ratio. **(C)** Hematoxylin and eosin staining of the slices of the RV and **(D)** of the cardiomyocyte area of the RV. The values are the mean ($\bar{\text{x}}$) ± standard error (SE). ^∗^*p* < 0.05: hypoxia-exposed vs. NX; ^#^*p* < 0.05: CIH vs. CH. ^†^*p* < 0.05: AL vs. CR.

RVH was observed in both the AL and CR groups under hypobaric hypoxic conditions compared to the NX groups (*p* < 0.01) and was higher in the CH groups than in the CIH groups. Remarkably, a lower degree of hypertrophy was observed in the CR group than in the AL group (*p* < 0.05), and the degree of hypertrophy observed in the CR group under CH was similar to that in the AL group under CIH (Figure 2A). Similar results were found with total ventricles weight vs. body weight ratio (Figure 2B). Representative images and the quantification of the area clearly show the enlargement of myocytes, which was coincident with the hypertrophy found in the CR and AL groups (Figures 2C,D). Interestingly, the weights of both ventricles (RV and LV) in rats of the AL group were higher than those in the CR group, as expected.

### AMPK, mTOR, and PP2C Expression Levels

**FIGURE 3:**
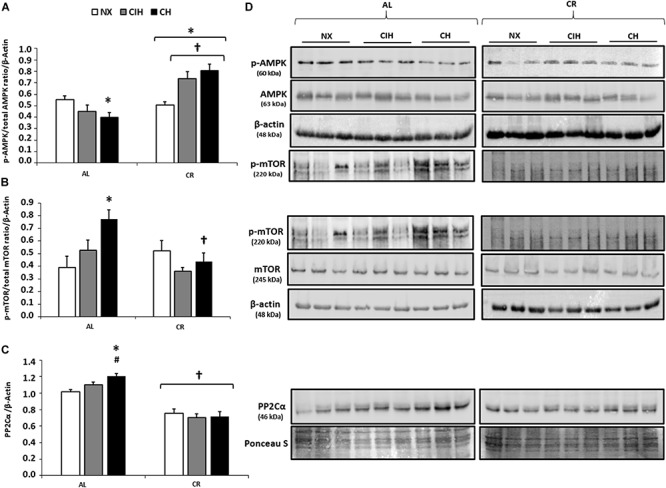
Protein expression levels in the right ventricle (RV): Ad libitum (AL) and caloric restriction (CR) groups at day 30: normoxic group (NX), chronic intermittent hypoxia group (CIH), and chronic hypoxia group (CH). **(A)** Activation of AMPK (p-AMPK/total AMPK ratio); **(B)** Activation of mTOR (p-mTOR/total mTOR ratio) normalized by β-actin; **(C)** PP2Cα expression levels, normalized by Ponceau staining; and **(D)** Representative bands are shown. Values are means (X) ± standard errors (SEs). ^∗^*p* < 0.05: hypoxia-exposed vs. NX; ^#^*p* < 0.05: CIH vs. CH. ^†^*p* < 0.05: AL vs. CR.

AMPK activation was increased only in the CR groups under both CIH and CH (*p <* 0.05), while the AL groups showed no AMPK activation. Interestingly, in this latter group, a decrease in AMPK under CH was found (*p* < 0.05) (Figures 3A,D).

Conversely, in the CR groups under both CIH and CH, there was no mTOR activation and no overexpression of PP2Cα, while in the AL group, there was a higher activation and expression of both proteins under CH than under CIH (*p <* 0.05) (Figures 3B–D).

## Discussion

This research on long-term CIH and CH right ventricular hypertrophy revealed important and novel findings. Caloric restriction results in: (1) reduced RVH; (2) activation of AMPK; and (3) no activation of mTOR and PP2Cα was seen.

This research is in line with other studies and calls attention to the importance of a low body weight to decrease cardiac risk (Riordan et al., 2008; Derumeaux et al., 2017) with ventricular hypertrophy (Karwi et al., 2019). However, the influence of body weight on AMPK activation in RVH induced by CH and long-term CIH exposure has received little attention.

In this study, both the AL and CR groups lost body weight and had lower blood glucose and serum insulin levels due to the metabolic effects of hypoxia, as has been reported with hypobaric and hypoxic hypoxia models (Chiu et al., 2004; Gamboa et al., 2011; Siques et al., 2018). Moreover, the CR group not only lost body weight under hypoxia but also began exposure with a lower body weight than did the AL group. These results call attention to the importance of maintaining a low body weight before and during exposure to long-term hypobaric hypoxia.

Interestingly, regarding hematocrit, the CR group had a lower hematocrit before the exposure, with no difference among the groups. This result is consistent with those of some authors, who explain that this lower hematocrit is the effect of decreased intake of dietary energy and of blood-forming nutrients (Faris et al., 2012; Gasmi et al., 2018). Despite the possible influence of CR in the hematocrit level, it is important to highlight that the all exposed to hypoxia groups had elevated hematocrit at day 30.

On the other hand, the model of this study is effectively an unusual model of exposure that is scarcely known. Food intake clearly has an effect on insulin level, but the regimen and degree of hypoxia are also extremely important, which is in agreement with the results of Debevec and Millet (1985), who demonstrated that the regimen and the degree of hypoxia can lead to variation in the results. In our results, the degree of hypoxia was the most important variable because it determined the amount of food intake, although the difference was not significant between the CR and the AL in CH groups. Interestingly, AL groups, in both hypoxia conditions show a decrease of food-intake and a possible explanation could be attributed to the hypoxic stressor and its side effects (San Martin et al., 2017).

Conversely, the CIH groups eat differently under hypoxic stimuli. Those fed ad libitum eat 4.5 g; however, the CR group eat an average of 7.9 g. The AL groups could have been affected by anorexia and general malaise, as occurs in humans (acute mountain sickness), and particularly so when just slightly over a healthy body weight (as described by San Martin et al., 2017). Furthermore, the AL group maintained a substantial amount of fat tissue, which has an influence in insulin. Additionally, insulin sensitivity has been demonstrated to be increased in hypoxia (Chiu et al., 2004; Gamboa et al., 2011). Therefore, both food intake and hypoxia regimen would contribute to determine insulin levels, highlighting that the kind and degree of hypoxia might be the most critical.

Ventricular hypertrophy) is characterized by increased cardiomyocyte size, a higher degree of sarcomere organization and enhanced protein synthesis levels, all of which are closely associated with energy metabolism (Tham et al., 2015). Human studies in the same model of CIH showed that this hypertrophy is associated with cardiometabolic factors, among others (Brito et al., 2018). Our results demonstrate RVH in those exposed to hypoxia, and importantly, that RVH in CR is lesser than in AL. Although, in this study does not seems to be restricted to the latter, because CR rats eat more food than AL inside the chamber in CIH condition; and the CR rats did developed RVH as well but at a lesser, only under hypoxic conditions, where AMPK pathway might play a major role as is discussed below. However, in the current study, it is clear that with CR, the hypoxia regimen increased the AMPK activity and inhibited mTOR, which might explain the degree of RVH that was found. The weights of both ventricles, including LV, in rats of the AL group were higher than those in the CR group, as expected because of the pre-exposure weight.

AMPK is known as an energy sensor and a regulator of cardiac energy metabolism under normal and stress conditions. Additionally, AMPK has been shown to inhibit cardiac hypertrophy (Sung et al., 2015) and to have a role beyond metabolic regulation. It also plays a critical role in an ample variety of cellular processes, such as regulating protein synthesis, transcriptional activity and energy supply (Baskin and Taegtmeyer, 2011; Cates et al., 2018; Feng et al., 2018). Additionally, CR-induced relative energy deficits, such as hypoxia, hypertrophy and hypoglycemia, result in increased intracellular levels of the AMP/ATP ratio, activating AMPK (Arad et al., 2007; Viollet et al., 2009). Our study (caused by pressure overload as a consequence of hypoxia) presents all of the above stressors; however, only CR groups that began with a lower body weight prior to exposure and maintained it exhibited the activation of AMPK activation and lower RVH. Similarly, other studies demonstrate other pathways that involve AMPK, showing nutrient deprivation produce an increase of AMPK activity, and this activation decreases the cardiac remodeling through degradation of hypertrophic proteins mediated by AMPK-induced transcription factors, such as MEF2 (Baskin and Taegtmeyer, 2011). However, in our study the hypoxic stressor appears to play a clear role. Another kind of RVH induction in rats showed that the activation of AMPK by metformin significantly reduced RVH, highlighting the importance of AMPK in clinical treatments for RVH (Li et al., 2016). Other evidence has demonstrated that pharmacological AMPK activation attenuates the development of cardiac hypertrophy by inhibiting protein synthesis through the inactivation of the mammalian target of rapamycin (mTOR) signaling pathway (Chan et al., 2004, 2008; Kang et al., 2011).

Thus, in the process of regulating ventricular hypertrophy mTOR is involved. mTOR is the key sensor of nutrient status, consisting of two distinct complexes, mTORC1 and mTORC2, and its activation contributes to cell survival in cardiomyocytes and regulates cell proliferation, apoptosis, cell migration and metabolism. Moreover, the protective effects of AMPK on PO-induced cardiac hypertrophy were recently shown to be partially mediated by the inhibition of mTORC1 signaling but not mTORC2 signaling (Li et al., 2014). This study, focused on mTORC1, showed that only the CR group demonstrated no activation of mTOR, which supports the role of the activation of AMPK as a protector against RVH in high-altitude hypoxia (hypobaric hypoxia).

Conversely, in the AL group exposed to CH, increased mTOR activation and decreased AMPK activation showed an inverse regulation between the two kinases. Further support for our findings comes from reports demonstrating that the inhibition of mTOR under chronic hypobaric hypoxic conditions resulted in the prevention of RVH in an animal model (Paddenberg et al., 2007) and that increased AMPK activity and decreased mTOR activity attenuated RVH in pulmonary-artery-hypertension-induced rats (Deng et al., 2017).

In this study, we have shown that the modification of body weight through diet leads to reduced RVH under high-altitude exposure. CR has been established as a potent dietary intervention that produces beneficial cardiac effects (Kobara et al., 2015; Melo et al., 2016) through AMPK activation, which plays an important role in cardioprotection, as has been demonstrated in the left ventricle (Shinmura et al., 2005, 2008; Chen et al., 2013).

On the other hand, it was interesting to observe that the PP2Cα expression level was increased in the AL group after CH exposure but not in the CR group. This finding further supports the lack of AMPK activation. Notably, the time of hypoxia exposure in the CIH group was lower than that in the CH group. This fact, could explain the reason to have a remarkably RVH in AL’s chronic groups.

Many authors have described that AMPK inactivation by dephosphorylation is attributed to PP2C (Davies et al., 1995; Marley et al., 1996; Steinberg, 2007). This dephosphorylation is enhanced under nutrient-rich conditions (the AL group of this study) but deranged under nutrient-poor conditions (Davies et al., 1995; Sanders et al., 2007; Hardie et al., 2012). The finding of a lack of expression of this phosphatase would be another way to explain the reduced hypertrophy in the CR group allowing AMPK activation.

The aim of this study was to evaluate RVH in different regimens of long-term hypoxia and not the degree of pulmonary hypertension, which is well known that hypoxemia induce an increase in pulmonary artery pressure and all the involved mechanisms leading to RVH. Given that we cannot correlate both proteins (AMPK and mTOR) with the actual degree of PAP at any stage, this would be a limitation. However, in that sense, the literature supports and describes that CR could decrease PAP and RVH (Ding et al., 2015). Thence, in the current study, it is clear that with CR, and the hypoxia regimen increased the AMPK activity and inhibited mTOR, which might explain the RVH that was found.

In addition, Wang and Unger (2005) suggested that AMPK activity was reduced and the expression of PP2C was increased significantly in the hearts of obese rats, which is rather in agreement with the histological findings of more fat between myocytes in the AL group, although the rats in these groups were not obese. This study also has the limitation that obese rats were not included, since the inclusion of obese rats raises the concern of introducing a bias, and the main aim was just to analyze CR in the development of RVH. Another limitation of this study is the lack of assessment of the cardiac functional status because the focus was more for cardiac morphology.

## Conclusion

This study contributes to a better understanding of the possible relationship between body weight and AMPK activation in the development of RVH under hypoxia. Caloric restriction, either under CH or CIH, would be contributory to a decreased degree of high-altitude-induced RVH through the activation of AMPK. Nonetheless, further research is necessary to corroborate this finding and whether it can be translated to clinical grounds.

## Data Availability Statement

All data sets for this study are included in the manuscript.

## Ethics Statement

The animal study was reviewed and approved by the Research Ethics Committee of Arturo Prat University, Iquique, Chile.

## Author Contributions

KF, PS, and JB conceived and designed the study, performed the experiments, analyzed and interpreted the data, drafted the manuscript, critically revised important intellectual content in the manuscript, and provided overall supervision. ÁL and SA contributed to the interpretation of the results and assisted in critical decisions and revision. FL-V, KA, SO, EP, and RL contributed to critical revisions of the manuscript. All authors approved the final manuscript and agreed to be accountable for all aspects of the work.

## Conflict of Interest

The authors declare that the research was conducted in the absence of any commercial or financial relationships that could be construed as a potential conflict of interest.
